# DNA methylation episignature for White-Sutton syndrome due to *POGZ* variants

**DOI:** 10.1016/j.gimo.2026.104489

**Published:** 2026-08-05

**Authors:** Sadegheh Haghshenas, Brittany N. Simpson, Amelle Shillington, Aurore Garde, Aidin Foroutan, Michael A. Levy, Raissa Relator, Pratibha Bhai, Jennifer Kerkhof, Haley McConkey, Frederic Tran Mau-Them, Ange Line Bruel, Christophe Philippe, Anne-Sophie Denommé-Pichon, Hana Safraou, Aline Vincent, Olivier Patat, Fabienne Prieur, Laurence Perrin, Christine Coubes, Bertrand Isidor, Melanie Fradin, Annick Toutain, Benoit Mazel, Paul Kuentz, Julian Delanne, Christel Thauvin-Robinet, Clément Simao De Souza, Caroline Racine, Giuseppe Merla, Marion Gerard, Nicolas Gruchy, Cindy Colson, Marielle Alders, Saskia Maas, Matthew L. Tedder, Wesley G. Patterson, Emanuela Salzano, Emi Rikeros Orozco, Sixto García Miñaúr, María Palomares-Bralo, Antonio Vitobello, Bekim Sadikovic, Laurence Faivre

**Affiliations:** 1 Verspeeten Clinical Genome Centre, London Health Sciences Centre, London, ON, Canada; 2 Cincinnati Children's Hospital Medical Center: Division of Human Genetics, United States; Cincinnati Children's Hospital Medical Center: Department of Pediatrics, University of Cincinnati College of Medicine, Cincinnati, OH; 3 Université Bourgogne Europe, CHU Dijon Bourgogne, Inserm, CTM UMR1231, équipe GAD, FHU TRANSLAD, Centre de génétique, Centre de référence Anomalies du Développement et Syndromes Malformatifs, Centre de référence Déficiences In-tellectuelles de Causes Rares, et Centre de référence GénoPsy, Dijon, France; 4 Department of Pathology and Laboratory Medicine, Western University, London, ON, Canada; 5 Université Bourgogne Europe, CHU Dijon Bourgogne, Laboratoire de Génomique Médicale, Centre Neomics, FHU TRANSLAD, Centre de recherche Translationnelle en Médecine moléculaire – Inserm UMR1231, équipe GAD, Dijon, France; 6 Centre de Référence Anomalies du Développement et Syndromes Malformatifs, CHU Caen, Caen, France; 7 Centre de Compétence Anomalies du Développement et Syndromes Malformatifs, CHU Toulouse, Toulouse, France; 8 Centre de Compétence Anomalies du Développement et Syndromes Malformatifs, CHU Saint Etienne, Saint Etienne, France; 9 Centre de Référence Anomalies du Développement et Syndromes Malformatifs, Hôpital Robert Debré, APHP, Paris, France; 10 Centre de Référence Anomalies du Développement et Syndromes Malformatifs, CHU Montpellier, Montpellier, France; 11 Centre de Référence Anomalies du Développement et Syndromes Malformatifs, CHU Nantes, Nantes, France; 12 Centre de Référence Anomalies du Développement et Syndromes Malformatifs, CHU Rennes, Rennes, France; 13 Centre de Référence Anomalies du Développement et Syndromes Malformatifs, CHU Tours, Tours, France; 14 Oncobiologie Génétique Bioinformatique, FHU TRANSLAD, Institut GIMI, CHU Besançon, Besançon, France; 15 Laboratory of Regulatory and Functional Genomics, Fondazione IRCCS Casa Sollievo della Sofferenza, San Giovanni Rotondo (Foggia), Italy; 16 Department of Molecular Medicine and Medical Biotechnology, University of Naples Federico II, Via S. Pansini 5, 80131 Naples, Italy; 17 Medical Genetics Department, Caen University Hospital, Caen F-14033, France; 18 CHU Lille, Clinique de Génétique Guy Fontaine, Centre de Référence Maladies Rares "Anomalies Du Développement et Syndromes Malformatifs" Nord-Ouest, Lille, France; 19 Department of Human Genetics, Amsterdam Reproduction & Development Research Institute, Amsterdam University Medical Centers, University of Amsterdam, Amsterdam, The Netherlands; 20 Greenwood Genetic Center, Greenwood, SC; 21 Medical Genetics Unit, AOOR Villa Sofia-Cervello Hospitals, Palermo, Italy; 22 Institute of Medical and Molecular Genetics (INGEMM), Hospital Universitario La Paz, IdiPAZ, CIBERER, ISCIII, Madrid, Spain

**Keywords:** DNA methylation, Epigenetics, *POGZ*, WHSUS, White-Sutton syndrome

## Abstract

**Purpose:**

White-Sutton syndrome (WHSUS) is a rare, autosomal dominant neurodevelopmental disorder (NDD) caused by heterozygous loss-of-function alterations in the *POGZ* gene. WHSUS exhibits variable expressivity, often overlapping with other NDDs. In this study, we characterized a distinct DNA methylation epigenetic signature (episignature) that differentiates WHSUS from unaffected individuals, as well as from individuals with other NDDs that also have episignatures, using a cohort of both published and unpublished cases of *POGZ* haploinsufficiency.

**Methods:**

Thirteen DNA samples from the peripheral blood of individuals with pathogenic or likely pathogenic heterozygous alterations in *POGZ* were used to identify the episignature and develop a predictive diagnostic biomarker. The diagnostic model, validated with 4 samples from confirmed WHSUS cases, was then applied to investigate the methylation patterns in 7 individuals with variants of uncertain significance in *POGZ*.

**Results:**

Using genome-wide DNA methylation analysis, a predominantly hypomethylated DNA methylation profile specific to WHSUS was identified. A sensitive and specific classifier model was then constructed based on this profile. Gene set enrichment analysis revealed terms related to various cellular processes, and functional correlation analysis indicated potential common pathways with other genetic disorders.

**Conclusion:**

We identified a novel and robust episignature in WHSUS caused by *POGZ* haploinsufficiency. This episignature facilitates the identification and diagnosis of individuals with WHSUS, as well as the reclassification of variants of uncertain significance associated with this condition.

## Introduction

Pathogenic variants in genes encoding the epigenetic machinery responsible for reading, writing, or erasing posttranslational signals on DNA and histone proteins are implicated in a wide range of constitutional neurodevelopmental disorders (NDDs). One such condition is White-Sutton syndrome (WHSUS, OMIM 616364), a rare developmental disorder caused by heterozygous pathogenic variants in *POGZ* (Pogo transposable element-derived protein with zinc finger domain, HGNC:18801). The main features observed in WHUS include developmental delay, intellectual disability (ID), and neurobehavioral abnormalities, such as autism spectrum disorder (ASD).

The disorder can also be referred to as the intellectual disability-microcephaly strabismus-behavioral abnormalities syndrome (MONDO:0014606). Additional features may include hypotonia, sensorineural hearing impairment, visual defects, a tendency to be overweight, gastrointestinal difficulties, and dysmorphic facial features.^1^, ^2^, ^3^, ^4^, ^5^ Despite some clinical similarities, there is significant phenotypic heterogeneity even among individuals with similar types of *POGZ* variants.^3^ The *POGZ* variants reported as pathogenic in ClinVar (https://www.ncbi.nlm.nih.gov/clinvar/) in individuals with NDDs include nonsense, frameshift,^2^ splice site, and missense variants.^4^ These variants are most often *de novo*, but familial cases have been reported with mild clinical features.^1^^,^^2^ *POGZ* is one of the genes most commonly associated with recurrent *de novo* variants in NDDs,^6^ including ASD and ID.

Some authors have explored genotype–phenotype correlations in individuals with *POGZ*-associated NDDs, aiming to understand how specific types and locations of variants influence the clinical spectrum. A comparative analysis of mild versus severe phenotypes has been conducted with respect to the type of variant (missense or truncating), its location within the *Pogz* protein, and the predicted ability of the transcript to undergo nonsense-mediated RNA decay (NMD) in modulating disease severity.^2^ The findings suggest a significant correlation between missense variants and milder clinical presentations, whereas truncating variants predicted to escape NMD are more frequently associated with severe phenotypes. Among these, truncating variants located in the proline-rich domain of the *Pogz* protein correlate with the most severe manifestations.*2* These observations support the hypothesis that gain-of-function or dominant-negative effects resulting from NMD escape, along with the specific location of the variant within critical functional domains, notably the proline-rich region, may be key determinants of the clinical severity of *POGZ*-related disorders.

*POGZ* is constitutively expressed in most tissues, with especially strong expression in the human fetal and adult central nervous system, including the cerebral cortex, cerebellum, spinal cord, hippocampus, and basal ganglia.^6^^,^^7^ *POGZ* is co-expressed with other genes associated with autism risk that are involved in chromatin remodeling and transcriptional regulation in the human fetal brain.^8^^,^^9^ These expression profiles highlight the critical roles of *POGZ* during human cortical development. Furthermore, recent studies suggest that *POGZ* regulates the neural differentiation of mouse neural stem cells through chromatin-silencing pathways (7). POGZ interacts with isotypes of the human heterochromatin protein 1 (HP1), which was first described in *Drosophila* as an essential component of constitutive heterochromatin required for stable epigenetic gene silencing^10^^,^^11^ A knockout mouse model has demonstrated that *Pogz* is involved in transcriptional repression and the dysregulation of multiple genes related to neuronal development and function. In particular, severe alterations in gene expression in the cerebellum were accompanied by changes in neuronal activity.^12^ Together, these findings indicate that *POGZ* is an integral component of heterochromatin-binding protein complexes that are essential for cell cycle progression and gene regulation during human cortical development.

An increasing number of rare diseases associated with chromatin regulatory genes are being linked to unique genome-wide methylation patterns, known as episignatures, which are detectable in the peripheral blood of affected individuals.^13^ Episignatures have proved to be sensitive and specific biomarkers for patients with ambiguous clinical or genetic findings.^14^, ^15^, ^16^, ^17^, ^18^

Based on these data, we hypothesized the presence of a DNA methylation episignature for WHSUS. In the present study, we report a specific DNA methylation signature in peripheral blood specimens from individuals with pathogenic or likely pathogenic variants in *POGZ*, distinct from those of other diseases with known episignatures.

## Materials and Methods

### Study cohort

Twenty-four individuals with pathogenic or likely pathogenic variants, as well as variants of uncertain significance (VUS), were recruited through international collaborations. Fifteen participants were from France, four from the USA, three from Spain, one from Italy, and one from the Netherlands). All samples were anonymized, and informed consent for the use of clinical information was obtained from all participants. Genotypic (hg19, Refseq: NM_015100.4) and clinical information for the participants are included in Tables 1 and 2.

**Table 1:** Information of individuals with *POGZ* variants who took part in this episignature study

Individual ID	Sex	Age (years)	Genomic Change (hg19; NC_000001.10)	DNA Change NM_015100.4	Protein Change NP_055915.2	Expected NMD Effect	MVP Score	Label
P1	M^a^	11	g.151402189T>C	c.460-2A>G	p.?	NMD	0.87	Discovery
P2	M	5.5	g.151396030G>C	c.1524-3C>G	p.?	NMD	0.87	Discovery
P3 (father of P2)	M	40	g.151396030G>C	c.1524-3C>G	p.?	NMD	0.95	Discovery
P4	M	4	g.151379389del	c.2545+1del	p.?	Escape NMD	1	Discovery
P5	F	6	g.151378676del	c.2836del	p.(Asp946MetfsTer12)	Escape NMD	1	Discovery
P6	M	3	g.151378740_151378753dup	c.2758_2771dup	p.(Gln925ProfsTer8)	Escape NMD	1	Discovery
P7	M	4	g.151384851dup	c.1702dup	p.(Ala568GlyfsTer3)	NMD	0.88	Discovery
P8	F	13.5	g.151378804_151378807dup	c.2704_2707dup	p.(Pro903HisfsTer42)	Escape NMD	0.99	Discovery
P9	M	12.5	g.151378390G>A	c.3121C>T	p.(Gln1041Ter)	Escape NMD	0.87	Discovery
P10	F	19	g.151379481G>T	c.2451C>A	p.(Cys817Ter)	NMD	0.88	Discovery
P11	F	7	g.151378393C>T	c.3118G>A	p.(Glu1040Lys)	NA	0.87	Discovery
P12	F	11	g.151378305_151378306del	c.3206_3207del	p.(Tyr1069Ter)	Escape NMD	0.89	Discovery
P13	F	13	g.151378865_151378866del	c.2646_2647del	p.(Asn882LysfsTer14)	Escape NMD	1	Discovery
P14	F	7	g.151378192A>C	c.3319T>G	p.(Phe1107Val)	NA	0.9	VUS
P15	M	3	g.151395941C>T	c.1610G>A	p.(Arg537His)	NA	0	VUS
P16	M	27	g.151402146C>G	c.501G>C	p.(Met167Ile)	NA	0	VUS
P17	F	18	g.151381039G>A	c.2080C>T	p.(Arg694Ter)	NMD	0.97	Validation
P18	M	11.6	g.151380943G>T	c.2176C>A	p.(Pro726Thr)	NA	0.31	VUS
P19 (father of P18)	M	34.2	g.151380943G>T	c.2176C>A	p.(Pro726Thr)	NA	0.6	VUS
P20	M	44	g.151377876_151377877del	c.3637_3638del	p.(Trp1213AlafsTer31)	Escape NMD	0.96	Validation
P21	F	14.9	g.151377546G>A	c.3965C>T	p.(Ser1322Phe)	NA	0.37	VUS
P22 (mother of P21)	F	47.4	g.151377546G>A	c.3965C>T	p.(Ser1322Phe)	NA	0.57	VUS
P23	F	21	g.151380601G>A	c.2350C>T	p.(Arg784Ter)	NMD	0.4	Validation
P24	M	12	g.151378390G>A	c.3121C>T	p.(Gln1041Ter)	Escape NMD	0.76	Validation

*M*: male; *F*: female; *NA*: not applicable; *P15* and *P16*: VUS with nonconcordant signature; *NMD*: nonsense-mediated mRNA decay.

**Table 2:** Clinical features and molecular data of 24 individuals harboring POGZ variants

	Patients with Pathogenic, Likely Pathogenic or VUS Reclassified as Likely Pathogenic												
	Patient 1	Patient 2	Patient 3 (father of P2)	Patient 4	Patient 5	Patient 6	Patient 7	Patient 8	Patient 9	Patient 10	Patient 11	Patient 12	Patient 13
**Country of origin**	Italy	France	France	France	France	France	France	France	France	France	France	France	France
**Variation**	c.460-2A>G, p.?,	c.1524-3C>G, p.?	c.1524-3C>G, p.?,	c.2545+1del, p.?	c.2836del, p.(Asp946MetfsTer12)	c.2758_2771dup, p.(Gln925ProfsTer8)	c.1702dupG, p.(Ala568GlyfsTer3)	c.2704_2707dup, p.(Pro903AsnfsTer42)	c.3121C>T, p.(Gln1041Ter)	c.2451C>A, p.(Cys817Ter)	c.3118G>A, p.(Glu1040Lys)	c.3206_3207del, p.(Tyr1069Ter)	c.2646_2647del, p.(Asn882LysfsTer14)
**Variant classification and criteria**	ACMG 1: PVS1 (±1 or 2 splice site); PM2 (Absent from control database gnomad); PM6 (de novo)	ACMG 2: PM2 (Absent from control database gnomad); PP3 (computational evidence support a deleterious effect); PP1-supporting (inherited from symptomatic father) PM6: Reported de novo in an affected individual	ACMG 2: PM2 (Absent from control database gnomad); PM6 (de novo); PP3 (computational evidence support a deleterious effect); PP1-supporting (Reported in another affected individual and inherited from symptomatic father)	ACMG 1: PVS1 (±1 or 2 splice site); PM2 (Absent from control database gnomad); PM6 (de novo)	ACMG 1: PVS1 (Frameshift variant, expected loss of function; PM2 (Absent from control database gnomad); PM6 (de novo)	ACMG 1: PVS1 (Frameshift variant, expected loss of function; PM2 (Absent from control database gnomad); PM6 (de novo)	ACMG 1: PVS1 (Frameshift variant, expected loss of function; PM2 (Absent from control database gnomad); PM6 (de novo)	ACMG 1: PVS1 (Frameshift variant, expected loss of function; PM2 (Absent from control database gnomad); PM6 (de novo)	ACMG 1: PVS1 (Nonsense variant expected loss of function); PM2 (Absent from control database gnomad); PM6 (de novo)	ACMG 1: PVS1 (Nonsense variant expected loss of function); PM2 (Absent from control database gnomad); PM6 (de novo)	ACMG 2: PM2 (Absent from control database gnomad); PM6 (de novo); PP3 (computational evidence support a deleterious effect); PS3-supporting (Reported de novo in affected individuals with some functional evidence (PMID: 26739615, 32103003, 25694107)	ACMG 1: PVS1 (Nonsense variant expected loss of function); PM2 (Absent from control database gnomad); PM6 (de novo)	ACMG 1: PVS1 (Frameshift variant, expected loss of function PM2 (Absent from control database gnomad); PM6 (de novo)
**Inheritance**	*de novo*	Paternal (symptomatic)	*de novo*	*de novo*	*de novo*	*de novo*	*de novo*	*de novo*	*de novo*	*de novo*	*de novo*	*de novo*	de novo
**Age at last examination**	11y	8y6m	ND	10y8m	6y	6y	3y 6m	12y4m	12y2m	18y11m	8y9m	12y4m	17y
**Sex/gender**	M	M	M	M	F	M	M	F	M	F	F	F	F
**Birth measurements**	BW 3370 g; BL 51 cm; OFC 34,5 cm at term	BW 3650g; BL 50cm; OFC 34cm at term	ND	ND	BW 3290g; BL 49 cm; OFC 33 cm at term	BW 2700g; BL 48cm; OFC 34cm at term	BW 3770g; BL 50cm; OFC 34cm at term	BW 1910g; BL 44cm; OFC 30cm et term	BW 3365g; BL 48cm; OFC 34cm at term	BW 950g, BL ND at 25 WG	W 3710 g; BL 48 cm; OFC 33.5 cm at 37 WG	BW 2840g; BL 47cm; OFC 33cm at 36 WG	BW 2690g; BL 47.5cm; OFC 32.5cm
**Measurements at last examination (age)**	At 11 years, W 50 kg (95th centile); H 143 cm (55th centile), OFC 52.2 cm (10th centile)	At 8y6m, W 37.7 kg (+2.7SD); H 136.5 cm (+1.7SD); OFC 51.8 cm (-0.8SD); BMI: 20.2 kg/m² (>+2SD)	ND	At 10y8m, W 26.6kg (3rd centile); H 132.5cm (-1.5SD)	At 6y, W 21.2Kg (mean); H: 114cm (-0.5SD) OFC: 47.5 cm (-2.5SD)	At 6 y, W 19.5kg (+ 0.5SD); H 110cm (- 0.5SD); OFC 50cm (- 1.5SD)	At 3y6m, W 16.8kg (+1SD); L 100cm (mean); OFC 48.5cm (-2SD)	At 12y4m, W 30kg (-1.6SD); H: 137cm (-2.1SD); BMI: 16.0 (-0.9SD); At 9y5m, OFC 48cm (-3.2SD)	At 12y2m, W 53kg (+3SD); H 160cm (+2SD); OFC: 55cm (+0.5SD); BMI: 20,7 kg/m²	At 18y11m, W 75.5 kg (+3.6SD); H 162 cm (-0.2SD); OFC: 52.7 cm (-2SD); BMI: 28.8 kg/m²	At 8y9m, W 39,3kg (>+3SD); H 131.5cm (+1SD); OFC 50.5cm (-1SD); BMI: 22,7 kg/m2 ( +4SD)	At 12y4m, W 54 kg (97th centile); H 154 cm (mean); OFC 53.8 cm (mean); BMI: 22.6 (95th centile)	At 17y, W 62kg (75th centile); H 150cm (-2SD); OFC 53.5cm (-1SD)
**First words / Sentences**	First words: 30m; Sentences ND	First words: 3y; sentences ND	ND	No speech	No speech	No speech	3y/ND	No speech	2y / 5y	3y / ND	ND	12m / 5y	3y 3m / ND
**Walking age**	14m	18m	Delayed	6y	2.5y	2.6y	2y	Never acquired (as well as sitting position)	17m	24m	17m	18m	13m
**Learning disabilities**	Yes	Yes	Yes	Yes	Yes	Yes	Yes	Yes	Yes	Yes	Yes	Yes	Yes
**ID (severity)**	Mild-moderate ID	No ID	ND	Severe ID	Severe ID	Severe ID	ND	Severe ID	Borderline	Moderate ID	Mild ID	No ID	Severe ID
**IQ if available**	Leiter R IQ 56; fluid intelligence 68	ND	ND	ND	ND	ND	ND	ND	65 < QIT < 78	QIT 42	QIT 59	ND	ND
**Speech delay (type, severity)**	Linguistic decoding difficulties, phonetic disorder	Speech delay, at age 8y 6m, well-constructed sentences	ND	No speech	No speech	No speech	Speech delay	No speech, limited gestural communication	Mild expressive and receptive speech deficit	Severe speech delay	Short sentences, poor vocabulary, speech articulation difficulties	Delayed but good recovery with schooling and rehabilitation, normal speech at age 10 years	Speech delay
**Hyperactivity**	Yes	No	ND	Yes	Yes	Yes	Yes	No	No	No	No	No	No
**Anxiety**	No	Yes	ND	ND	No	No	No	ND	No	Yes	No	Yes	Yes
**Stereotypies**	Yes	No	Yes (hands)	No	Yes	Yes	No	No	No	No	No	Yes	Yes
**Aggressiveness**	No	Yes	ND	No	No	Yes	Yes	No	No	No	No	No	No
**Self-destructive behavior**	Yes	No	ND	Yes	No	No	No	No	No	No	No	No	No
**Social withdrawal**	Yes	No	ND	Yes	No	Yes	ND	Intermittent visual engagement	Yes	No	No	Yes	ND
**Other inappropriate behavior**	Oppositional-defiant disorder	Yes	Non-autonomous adult patient	Yes	Yes	Yes	No	ND	Attention deficit	Frustration intolerance, lack of autonomy	No	Limited interests, attention deficit, hpersensitivity to smells and textures	No
**Psychiatric Features / Schizophrenia**	No	No	ND	ND	ND	ND	No	ND	No	Yes (psychotic symptoms)	No	No	ND
**Sleep difficulties**	No	No	ND	Yes with sleep apnea syndrome	No	Yes	Yes	No	No	No	No	Severe sleep apnea	No
**Seizures**	No	No	ND	No	No	No	No	Epileptic encephalopathy, onset age 2y	No	No	No	No	No
**Neurological symptoms**	No	Walking with feet in internal rotation	ND	No	No	No	No	Dystonia during childhood, spastic tetra paresis with axial hypotonia and peripheral hypertonia, choreo-athetosic movements.	No	Brisk reflexes	No	Global hypotonia, motor slowness, dyspraxia, mild precarious balance	No
**Eye problems**	Hyperopia, astigmatism	Myopia	ND	Strabismus, hyperopia	Nystagmus, hyperopia, strabismus	Exophoria, hyperopia, astigmatism, narrowing of the visual field, micropapilla	No	External squint, tear ducts stenosis (resolved)	Myopia	Myopia, retinopathy	Myopia, astigmatism	No	No
**Hearing loss**	No	No	ND	Yes	No	No	No	Bilateral sensorineural hearing loss (60 dB left and 80 dB right)	No	No	No	No	No
**Gastro-intestinal manifestations**	Feeding difficulties in early infancy	No	ND	Oral sensory disorders, constipation, occlusion, cyclic vomiting	Constipation	Severe constipation	No	Constipation	No	ND	Constipation, occlusion	Oral disorders, frequent gag reflex	Vomiting
**Congenital malformations**	Cryptorchidism	No	ND	Common mesentary, atrial septal defect, micropenis, cryptorchidism	Double renal sinus	No	No	Atrial septal defect, talipes equinovarus	No	No	No	No	Left megauretere, right ureteral duplication with bilateral reflux
**Hypotonia**	No	No	ND	ND	Yes	Yes	No	Yes	No	ND	No	Yes	ND
**Tendance to obesity**	Yes	Yes	Yes	ND	No	No	No	No	Yes	Yes	Yes	Yes	ND
**Morphological features**	Synophrys, long eyelashes, high nasal root, prominent columella, short philtrum, pointed chin, short neck, toes abnormalities	No	No	Round face, telecanthus, epicanthus inversus, upslanted palpebral fissures, short nose, anteverted nostrils, downturned corners of the mouth, brachycephaly	Prognathism, everted vermillon of the upper lip, square-shaped face	Bluish sclera, downslanted palpebral fissures, hypertelorism, large forehead with a square chin, downturned corners of the mouth, flat profile, low set ears, thick helix	No	Microcephaly, synophrys, arched eyebrows, retraction of the middle level of the face, thin skin with apparent venous network, hypertrichosis, long hallux	Thick lips, large ears, upturned corners of mouth (frozen smile), mild retrognathia, pointed chin, anteverted nares	High-arched palate, 1 café-au-lait spot, elbow extension limitation with ulna valga, right gibbosity, stretch marks on the lower back	Synophrys, hypertrophy of the nasal bone, thin lips, dental diastasis, tapering fingers, café-au-lait spots, angioma, agenesis of a lower incisor	Wide nasal bridge, drooping cheeks, frozen smile, few expressive face, bradymetacarpia and flat feet	Hypertelorism, short palpebral fissures, pear-shaped nose
**Infection History**	No	No	ND	ND	Serous otitis	No	No	Multiple broncho-pulmonary infections	No	Multiple bronchiolitis	Chronic otitis media	No	ND
**Other features**	Hypothyroidism, migraines, atypical deglutition	Skin hyperlaxity	ND	ND	No	No	Autistic features	Intra uterine growth retardation and oligohydramnios, mild scoliosis, resolved umbilical hernia, immunological thrombocytopenic purpura complicated with Evans syndrome, died at 12y10m	No	Bronchopulmonary dysplasia (premature birth), asthma, scoliosis	Mild genu valgum	No	No
**Cerebral MRI**	ND	Not performed	ND	T2 and flair non specific punctiform hypersignals in white matter frontoparietal regions, wide ventricules	Olfactive bulbs hypoplasia, thin corpus callosum	Dandy Walker, cerebral atrophy, olfactive bulbs hypoplasia	ND	Global expansion of the ventricular system, pellucidum septum cyst, enlargement of the peri-cerebral spaces and cisterns	ND	Microcephaly, dysgenesis of the corpus callosum	Normal	Cerebellar atrophia	Vermian atrophy

	Patients with Pathogenic, likely pathogenic or VUS reclassified as likely pathogenic									Patients with VUS and Discordant Episignature	
	Patient 14	Patient 17	Patient 18	Patient 19 (father of P18)	Patient 20	Patient 21	Patient 22 (mother of P21)	Patient 23	Patient 24	Patient 15	Patient 16
**Country of origin**	France	Netherlands	USA	USA	USA	Spain	Spain	Spain	France	France	USA
**Variation**	c.3319T>G, p.(Phe1107Val)	c.2080C>T, p.(Arg694Ter)	c.2176C>A, p.(Pro726Thr)	c.2176C>A, p.(Pro726Thr)	c.3637_3638del, p.(Trp1213AlafsTer31)	NM_015100.4:c.3965C>T, NP_055915.2:p.(Ser1322Phe)	NM_015100.4:c.3965C>T, NP_055915.2:p.(Ser1322Phe)	NM_015100.4:c.2350C>T, NP_055915.2:p.(Arg784Ter)	NM_015100.4:c.3121C>T, NP_055915.2:p.(Gln1041Ter)	NM_015100.4:c.1610G>A, NP_055915.2:p.(Arg537His), VUS	NM_015100.4:c.501G>C, NP_055915.2:p.(Met167Ile), VUS
**Variant classification and criteria**	ACMG 3: PM2 (Absent from control database gnomad); PM6 (de novo); PP3 (computational evidence support a deleterious effect)	ACMG 2: PVS1 (Nonsense variant expected loss of function); PM2 (Absent from control database gnomad)	ACMG # 3: PM2 (Absent from control database gnomad), inherited from a symptomatic father, in silico scores were ambiguous	ACMG # 3: PM2 (Absent from control database gnomad)	ACMG 2: PVS1 (Frameshift variant, expected loss of function PM2 (Absent from control database gnomad)	ACMG # 3: PM2 (Absent from control database gnomad); PP3 (computational evidence support a deleterious effect), inherited from a symptomatic mother	ACMG # 3: PM2 (Absent from control database gnomad); PP3 (computational evidence support a deleterious effect)	ACMG 1: PVS1 (Nonsense variant expected loss of function); PM2 (Absent from control database gnomad); PM6 (de novo)	ACMG 1: PVS1 (Nonsense variant expected loss of function); PM2 (Absent from control database gnomad); PM6 (de novo)	ACMG # 3: PM2 (Absent from control database gnomad), inherited from an asymptomatic parent, in silico scores were ambiguous.	ACMG # 3: PM2 (Absent from control database gnomad), not inherited from mother, father not available, in silico scores were ambiguous.
**Inheritance**	de novo	ND (parents not tested)	Paternal	Unknown (Father of patient 18)	Non-paternal	Maternally inherited	ND	De novo	De novo	Maternal (asymptomatic)	ND (non-maternal, paternal sample not available)
**Age at last examination**	7y3m	19y	11y	36y	44y	14y	ND	24y	15y	3y4m	20y
**Sex/gender**	F	F	M	M	M	F	F	F	M	M	M
**Birth measurements**	BW 3740g; BL 51cm; OFC 34cm at 37WG	Normal birth parameters at 40WG	BW 2920g at 39WG	ND	ND	Within normal range	ND	ND	BW 3365g; BL 48cm; OFC 34cm	BW 1290g; BL 41cm; OFC: 27.5cm at 32WG	BW 879g; BL 30.5cm at 25WG
**Measurements at last examination (age)**	At 7y3m, W 28kg (80th centile), H 128.5cm (+1SD); OFC 49cm (-2SD)	At 19y, H 164.2 cm (+0.5SD)	At 11y, W 46kg (+1SD); H 141 cm (mean); OFC 54.5cm (50th centile)	At 36y, W 73kg (75th centile), H 180cm (+0.5SD), OFC ND	At 44y, W 110 kg (99th centile); H 168.3cm (-1SD); OFC 58.5cm (+2SD)	At14y, W 67 Kg (90th centile), H 157 cm (30th centile), OFC 50cm (<1st centile)	ND	At 24y, H 159.5cm (25th centile); OFC 52 cm (3rd centile)	At 15y, W 69kg (95th centile), H 167cm (+0.5SD); OFC 56 cm (mean)	At 3y4m, W 14kg (-0.5SD); H 103cm (+2SD); OFC 51cm (+0.5SD)	At 20y, H 173cm (mean); W 77kg (+0.5SD); OFC 58cm (80thcentile)
**First words / Sentences**	ND / 5y4m	Delayed, two word sentences at age 4	Delayed	Delayed	Delayed	1y / 3y	Delayed	Delayed	2y / 3.5y	18m / ND	Delayed
**Walking age**	34m	18m	16m	Delayed	ND (was behind on most milestones)	15m	ND	ND	17m	17m	Delayed
**Learning disabilities**	Yes	Yes	Yes	Yes	Yes	Yes	Yes	Yes	Yes	Yes	Yes
**ID (severity)**	Mild ID	Mild ID	Moderate ID	Mild ID	Suspected	Yes (severity ND)	ND	Mild ID	No	Yes, ND	Mild ID
**IQ if available**	QIT 68	ND	50	ND	Performed when he was younger but no report available	ND	ND	51	NA	ND	QIT 53
**Speech delay (type, severity)**	Severe expressive and receptive speech deficit, articulation difficulties	Verbal with speech delay	Verbal with speech delay	Mild, patient fully verbal	ND	Delayed language development and articulation problems	ND	Delayed speech and language development, expressive language difficulties	Delayed speech that necessitated speech therapy for many years	Expressive deficit	Verbal with speech delay
**Hyperactivity**	No	No	Yes	ND	No	Yes	ND	No	No	ND	Yes
**Anxiety**	Yes	No	No	Yes	No	Yes (eating anxiety)	ND	Yes	Yes	ND	Yes
**Stereotypies**	Verbal stereotypies	No	No	No	No	No	ND	Yes	No	No	Yes
**Aggressiveness**	No	No	Yes	No	No	Yes	ND	No	No	No	Yes
**Self-destructive behavior**	No	No	Yes	No	No	Yes	ND	Yes	No	No	Yes
**Social withdrawal**	No (or only related to anxiety)	No	No	Yes	No	No	ND	No	Yes	ND	Yes
**Other inappropriate behavior**	No	No	Yes	No	No	No	ND	Obsessive disorders, phobias, migraine, fear, inhibition	Shy, inhibition	No	Yes
**Psychiatric Features / Schizophrenia**	No	No	No	Depression	No	No	ND	No	No	ND	Yes, schizophrenia
**Sleep difficulties**	No	No	Yes with obstructive sleep apnea	Sleep apnea	No	No	ND	Yes	No	No	No
**Seizures**	No	No	No (on AEDS for staring spells but normal EEGs)	No	No	No	ND	No	No	No	No
**Neurological symptoms**	Brisk and polykinetic reflexes in the lower limbs, (doubt about a bilateral Babinski sign)	No	No	migraine	Migraines	Autism spectrum disorder	ND	No	No	No	No
**Eye problems**	Amblyopia, optomotor disorder	No	Strabismus	No	Bilateral optic atrophy	Astigmatism and hypermetropia	ND	Hyperopia, without structural ocular anomalies.	Myopia	Lens ectopia, bilateral retinal detachment	No
**Hearing loss**	No	No	No	No	Occasional ringing; no decrease in hearing	No	ND	No	No	No	No
**Gastro-intestinal manifestations**	No	No	No	GERD	GERD	No	ND	Follow-up in the digestive department	No	No	Mild constipation, unintentional weight loss at age 19
**Congenital malformations**	No	No	Laryngeal cleft requiring repair glossoptosis	Laryngeal cleft	No	No	ND	NA	No	Aortic dilatation, pectus carinatum	No
**Hypotonia**	No	No	Yes, mild	No	No	No	ND	NA	NA	Axial, at birth only	No
**Tendance to obesity**	No	No	No	Yes	Yes	Yes	ND	NA	Yes	No	Yes (complicated by psychiatric medications)
**Morphological features**	Synophris, thin and slightly arched eyebrows, mild hypertelorism, long and thin fingers, clinodactyly of the 1st toes, flat valgus feet	Short philtrum, partial alopecia	Hypertelorism, downturned corners of mouth, anteverted nares	Not documented	None	Microcephaly, ascending oblique palpebral fissures, thin upper lip	ND	Hypertelorism, thin upper lip, smooth philtrum, mild scoliosis	High forhead, large eyes	Typical marfanoid syndrome with hyperlaxity, arachnodactyly, malar flattening, high-arched palate, hypertelorism, Ghent criteria >14/20, maternally inherited (Negative 26-genes Marfan panel)	Long face, prominent forehead, mild hypotelorism, thin upper lip
**Infection History**	No	No	Recurrent lung infections (complications of laryngeal notch)	No	None	No	ND	ND	No	Recurrent otitis	No
**Other features**	Hyperlaxity	Asthma	No	Scoliosis, kyphosis	ND	No	ND	Wilms tumor at age 2, suspected precocious puberty at age 8, increased body hair	ND	Asthma	No
**Cerebral MRI**	Not performed	ND	Mild prominence of the lateral ventricles and third ventricle, without evidence for hydrocephalus. Mild prominence of the sulci. Mild parenchymal volume loss/volume contraction	ND	ND	Normal	ND	Normal	ND	ND	Mild periventricular white matter volume loss, more prominent in the parietal regions. Prior intraventricular hemorrhage within the right lateral ventricle

### DNA methylation analysis

DNA samples extracted from peripheral blood underwent bisulfite conversion and were processed using Illumina Infinium methylation EPIC bead chip arrays, with methylation analysis performed according to the manufacturer’s protocol. The EPIC array covers >850,000 human genomic CpG sites, encompassing 99% of RefSeq genes and 96% of CpG islands. The obtained methylated and unmethylated signal intensities were imported into R 4.0.3 for analysis. Normalization followed the Illumina method with background correction using the minfi package.^19^ Probes located on the X and Y chromosomes, those with a detection *P* value >0.01, probes known to contain a SNP at or near CpG interrogation sites, or those known to cross-react with other genomic regions were removed to ensure that the observed differences between the two groups were solely based on methylation changes rather than other potentially confounding factors.^20^ After this initial probe filtering step, where necessary, the age of the DNA specimen was predicted using the wateRmelon package.^21^ Principal component analysis (PCA) was performed to observe the overall structure of the batches and identify outliers.

### Selection of matched control participants for episignature discovery

With a case-to-control ratio of 1:4, 52 age- and sex-matched control samples (samples without a *POGZ* variant or a clinical diagnosis of WHSUS) were selected from the EpiSign Knowledge Database (EKD),^13^ (https://episign.lhsc.on.ca/knowledge_database.html) using the MatchIt package^22^ as the control training set. PCA was conducted after each matching round to detect outliers and examine the data structure. Outlier samples and samples with an irregular data structure were removed at each iteration. This process was repeated until no outliers were observed in the first two principal components of the PCA.

### DNA methylation profiling

The probe selection procedure followed our previously published protocols.^13^^,^^16^ Methylation levels, represented as β values, were calculated for each probe as the ratio of methylated signal intensity to the sum of methylated and unmethylated signal intensities. These values were then transformed into M values using the logit transformation formula log_2_(β/(1-β)), ensuring homoscedasticity for use in linear regression. Using the limma package,^23^ we conducted linear regression, incorporating blood cell type compositions estimated using the algorithm developed by Houseman et al^24^ as confounding variables in the model matrix. The obtained *P* values were then moderated using the eBayes function and corrected for multiple testing using the Benjamini-Hochberg (BH) method. Probe selection was conducted through a multistep process designed to prioritize discriminatory power while minimizing redundancy and ensuring robustness against arbitrary parameter choices. Differentially methylated probes were first ranked using a composite score defined as the product of the mean methylation difference between the 2 groups and the −log10 of the BH-corrected *P* value. To evaluate robustness, we systematically tested different initial probe set sizes (800, 900, and 1000), varied the proportion of probes retained based on receiver operating characteristic curve performance (1/2, 1/3, 1/4, or 1/5 of the initial set), and applied different Pearson’s correlation thresholds for redundancy removal (0.60 to 1.0 in steps of 0.05). The combination of 900 initial probes, the top 450 probes selected based on the highest area under the receiver operating characteristic curve, and a correlation threshold of r > 0.85 provided the most robust separation of WHSUS cases and controls while minimizing redundant signals. After applying these filters, 212 nonredundant probes were retained to define the WHSUS episignature. The robustness of each probe set in distinguishing between case and control samples was assessed using unsupervised models. These models included hierarchical clustering performed using Ward’s method on Euclidean distance, as well as multidimensional scaling (MDS) based on scaling of the pairwise Euclidean distances between samples. Following this, 13 rounds of leave-one-out cross-validations were conducted. In each iteration, one of the 13 confirmed WHSUS samples was excluded, leaving 12 WHSUS samples and the 52 matched controls for probe selection. The selected probes from each iteration were then used to perform MDS. The WHSUS sample that was excluded in each iteration was plotted alongside the other case and control samples to observe whether it clustered with the case samples.

### Construction of the classification model

Using the selected probes, a binary support vector machine (SVM) was constructed using the e1071 package, as previously described.^16^^,^^17^ To determine the optimal hyperparameter (cost) and assess classifier accuracy, a 10-fold cross-validation was performed during training. In each round of 10-fold cross-validation, 90% of the samples were used for training, and the remaining 10% were used for testing. The model assigns a methylation variant pathogenicity (MVP) score ranging from 0 to 1 to each sample. Scores close to 1 indicate high similarity between the methylation profile of the sample and the identified episignature, whereas scores near 0 indicate low similarity. To confirm the model’s specificity, 2 SVM classifiers were generated. The first model was trained on 13 WHSUS case samples with pathogenic *POGZ* variants (individuals P1-P13) against matched control samples, supplemented with more than 1000 testing samples from other rare genetic disorders within the EpiSign v3 clinical classifier of the EKD^13^ to assess its specificity. The second classifier was trained on the same 13 individuals with *POGZ* pathogenic variants against matched control samples, with 75% of additional control samples and participants with other disorders from the EpiSign™ v3 clinical classifier within the EKD (Supplemental Table 3).^13^ The remaining 25% were used for testing the model. Variant classification was performed using a combined approach, integrating the SVM-derived MVP score with hierarchical clustering and MDS. Based on this integrated assessment, an MVP score threshold of 0.25 was empirically determined to capture all samples that cluster robustly with the WHSUS episignature.

### Functional correlation analysis

The subsequent analyses followed our previously published procedure.^25^ To assess the extent of overlap between the methylation profiles of WHSUS and other disorders from the EpiSign v3 clinical classifier within EKD,^13^ a set of 52 control samples was selected. These controls were matched to the case samples based on age and sex. Importantly, none of the selected control samples had been diagnosed with a disorder possessing a recognized episignature. This strategy for selecting control samples was driven by the goal of examining the similarity between the methylation patterns of WHSUS and those associated with other disorders known to have episignatures. Therefore, individuals with any of those specific disorders were excluded from the control set to ensure a more focused analysis. Since the differentially methylated positions (DMPs) selected for the disorders were found in either the 450 K array, the EPIC array, or both, only probes common to both arrays were considered.

To identify differentially methylated probes, the selection criteria included probes that showed a mean methylation difference >5% between WHSUS participants and control samples, along with BH-corrected *P* values < .01.

To demonstrate the degree of similarity between the DNA methylation patterns of WHSUS and other episignature disorders in the EKD included in the EpiSign v3 clinical classifier,^13^ we employed an agglomerative clustering approach, visualized through a tree-and-leaf diagram. For each cohort, we selected *n* DMPs with the lowest *P* values, where *n* represents the minimum value between 500 and the number of DMPs in that cohort. Subsequently, a β value matrix was created, with rows representing probes and columns representing cohorts. For each probe, the methylation value for a given cohort was determined as the median of the β values across all samples within that cohort. Using the resulting β matrix, we performed Euclidean clustering using Ward method. Finally, the outcome of the Euclidean clustering analysis was visualized in a tree-and-leaf diagram using the R package TreeAndLeaf version 1.6.1. A heatmap showing the degree of overlap between different cohorts was generated using the pheatmap package version 1.0.12.

To annotate DMPs and differentially methylated regions (DMRs), we used the R package annotatr, version 1.20.0,^26^ in conjunction with AnnotationHub, version 3.0.0. The annotations used included hg19_cpgs, hg19_basicgenes, hg19_genes_intergenic, and hg19_genes_intronexonboundaries.

### Differentially methylated regions

To identify DMRs, the study utilized the DMRcate package.^27^ DMRs were selected based on the following criteria: regions containing a minimum of 5 CpG sites within a 1 kb span, with at least a 10% methylation difference between the case and control groups, and a Fisher’s multiple comparison *P* value of less than 0.01.

### Gene set enrichment analysis

Gene ontology (GO) enrichment analysis of the DMPs was performed using the missMethyl package, version 1.36.0.^28^

## Results

The study comprised 24 individuals, with 13 males and 11 females (Table 1). Seventeen individuals (P1-P13, P17, P20, P23, and P24) had pathogenic or likely pathogenic variants in *POGZ* (HGNC:18801) and confirmed clinical diagnoses of WHSUS*,* all of these were *de novo* except for one familial case (Figure 1A).^29^ Clinical characteristics are provided in Table 2. Thirteen of the 17 clinically and genetically confirmed WHSUS participants (labeled as “Discovery” in Table 1) were included in probe selection and the construction of the classification model (participants P1-P13), while an independent set of 4 participants with WHSUS (labeled as “Validation” in Table 1) were used to validate the episignature. The remaining 7 samples, which had *POGZ* VUS from 5 families, were included as testing samples (labeled as “VUS” in Table 1). Eight of the study samples were previously reported.^3^ Variants were classified according to the American College of Medical Genetics and Genomics and the Association for Molecular Pathology (ACMG/AMP) guidelines,^30^ with the criteria detailed in Table 2. The *in silico* tools used for the classification of the missense variants were CADD, PolyPhen, GERP, Grantham, AlphaMissense, and SIFT (Supplemental Table 2; references are provided in the supplemental data). The potential of each truncating variant to escape or trigger NMD was evaluated using the NMDEscPredictor tool (https://nmdprediction.shinyapps.io/nmdescpredictor/).

**Figure 1:**
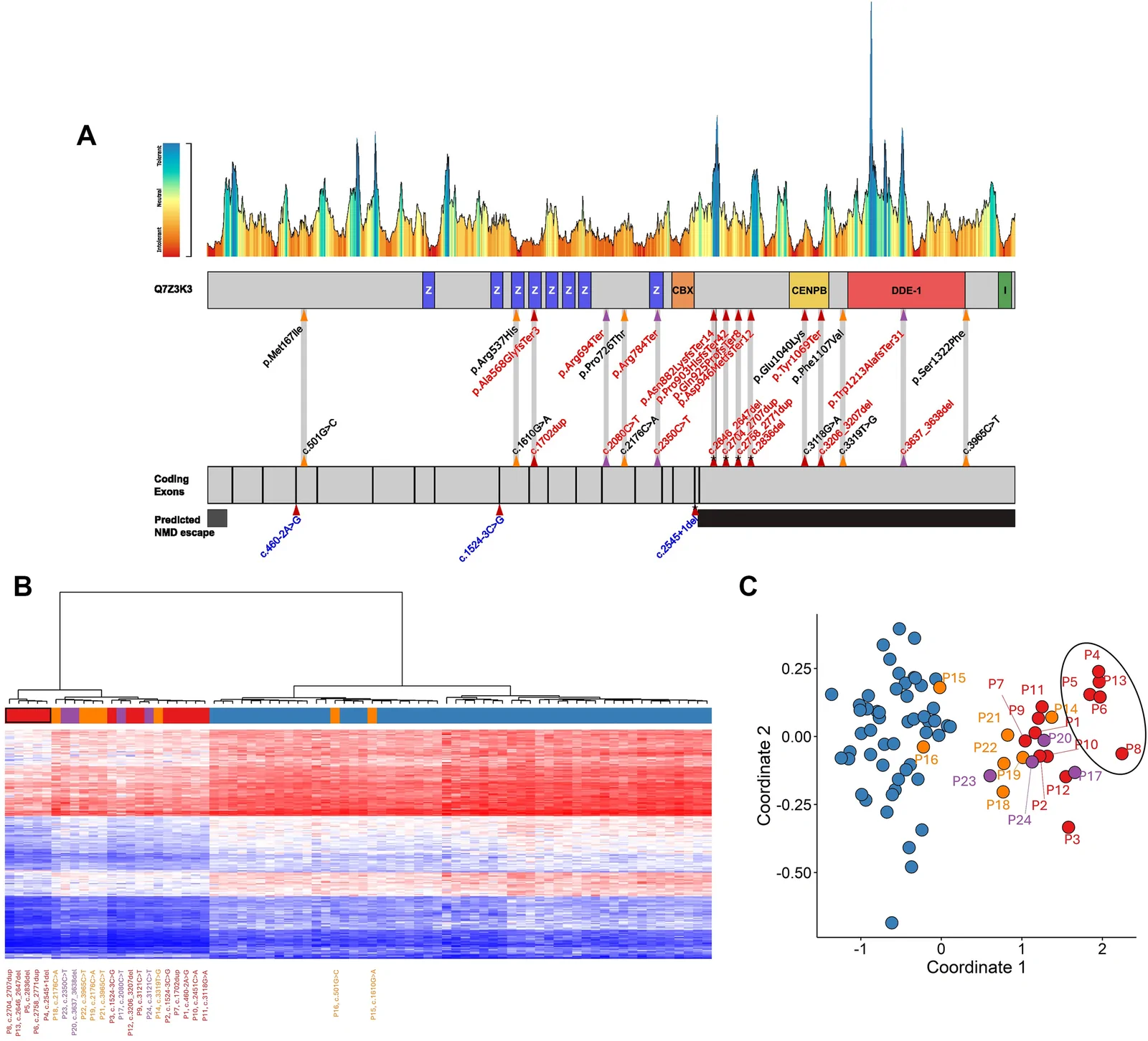
**Location of variants and incorporation of testing samples into the unsupervised models. (**A)** Mutational missense constraint obtained from MetaDome**^29^**is shown**. Exons, predicted nonsense-mediated mRNA decay (NMD) escape regions, and protein domains are depicted. Each arrow indicates the position of the variant identified in the respective participant (orange arrows: VUS; red arrows: pathogenic or likely pathogenic variants; purple arrows: validation samples). A black asterisk on the arrows indicates variants associated with a more distinct methylation difference relative to controls (as shown in panel **C**). Annotations and positions for Q7Z3K3 were obtained from UniProt. PR: polar residues, Z: C2H2 zinc finger domains; HPZ: region required for interaction with CBX5; Pro: composition bias proline residues; CENP: centromere protein CENPB-type binding domain; DDE: pongo-like transposase domain; CC: coiled-coil domain; I: integrase domain-binding motif. (**B)** Hierarchical clustering is shown, with each row representing one of the 212 selected probes and each column representing a sample. In the heatmap bar at the top, WHSUS samples are indicated in red, and control samples are indicated in blue. VUS and validation samples, used for testing only, are indicated in orange and purple, respectively. The heatmap pane shows methylation levels from blue (no methylation) to red (full methylation). (**C)** MDS plot using the same color scheme as panel A. Individuals P4, P5, P6, P8, and P13, who exhibited a more distinct methylation profile, are highlighted with solid black borders in both the heatmap and the MDS plots.

### Detection and verification of an episignature for WHSUS

DNA methylation profiling was conducted only on samples from individuals P1 to P13 and 52 matched control samples. The 212 selected probes (Supplemental Table 1) were used in clustering models to evaluate the robustness of the episignature in distinguishing case samples from controls (Supplemental Figure 1). Supplemental Figure 1 demonstrates a clear separation between case and control samples, verifying the robustness of the episignature. Additionally, individuals with variants in the region expected to escape NMD, near the proline-rich region (before the CENP domain), show a more distinct methylation difference relative to controls.

The selected probes were then used to plot the 4 validation samples and the 7 individuals with VUS alongside the 13 confirmed WHSUS case samples and the 52 matched control samples. This allowed us to observe whether they clustered with the case or control group (Figure 1B and C).^29^ Both plots demonstrate the clustering of validation samples with the case samples. Within the participants with VUS, individual P14, whose variant was *de novo*, and individuals P18, P19, P21, and P22, whose variants segregated with affected individuals, clustered with the case samples. Conversely, individuals P15 and P16 clustered with the control group.

The reproducibility of the episignature was assessed by conducting 13 iterations of leave-one-out cross-validation. In each round, using the selected set of probes, MDS was performed on 12 WHSUS samples, 52 control samples, and the left-out WHSUS sample, and the corresponding MDS plot was created. In all iterations, the test sample consistently clustered with the remaining WHSUS samples and was distinctly separate from the control samples, indicating a high reproducibility of the episignature (Supplemental Figure 2).

### Construction of the classification model

To classify WHSUS individuals with higher accuracy, 2 SVMs were constructed using the selected set of probes. The MVP scores generated by the 2 classifiers were plotted, and the results are shown in Supplemental Figure 3 and Figure 2. In Supplemental Figure 3, the majority of control samples and cases from other disorders received MVP scores <0.25, indicating the high specificity of the model. All validation samples received MVP scores >0.25, demonstrating the model’s high sensitivity. Two WHSUS samples with VUS received very low MVP scores, while 5 WHSUS samples received high scores. In Figure 2, all control samples and cases from other disorders received scores near 0, indicating the classifier’s full specificity. Individuals P4, P5, P6, P8, and P13 (indicated with a solid black boundary) received higher MVP scores. The 4 validation samples received MVP scores above the threshold of 0.25, indicating the model’s high sensitivity. Additionally, VUS participants P15 and P16 received very low MVP scores, while the remaining VUS samples received high scores, leading to the reclassification of these variants as pathogenic.

**Figure 2:**
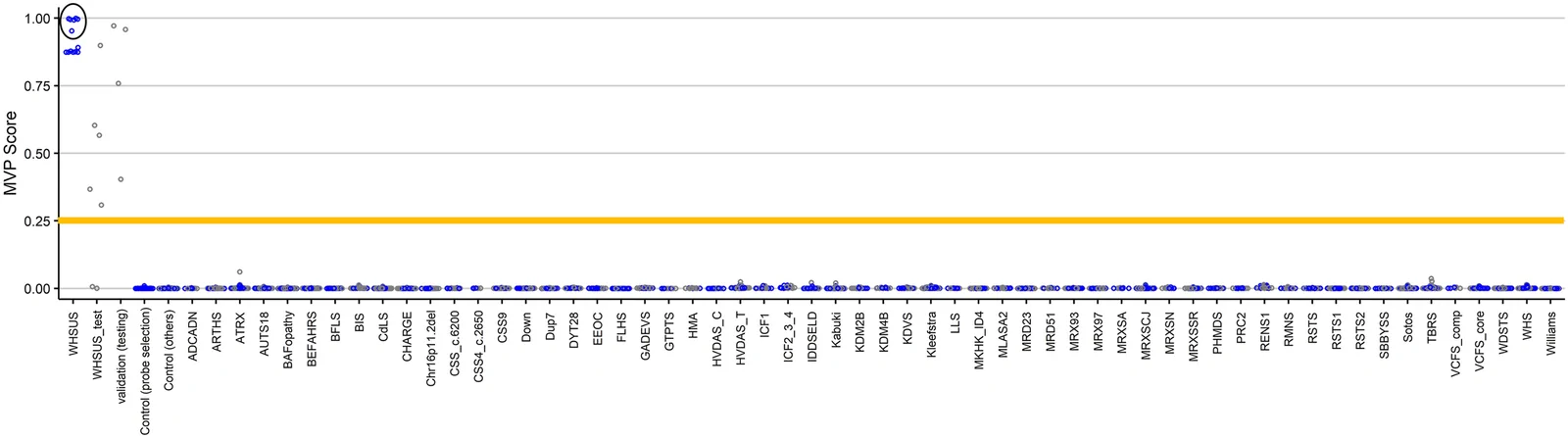
**MVP scores generated by the classifier obtained by training with 13 WHSUS samples (P1-P13) against control samples and cases from other syndromes**. Blue circles represent training samples, while gray circles represent testing samples. The 5 WHSUS training cohort samples, indicated with a solid black boundary, consist of individuals P4, P5, P6, P8, and P13. Supplemental Table 2 lists the EpiSign v3 disorders along with their abbreviations.

### Functional correlation analysis

Using the criteria described in the Materials and Methods section, 5450 DMPs and 16 DMRs were identified. These DMRs overlap several genes involved in transcription, the central nervous system, or cellular and tissue functions. The DMRs are listed in Supplemental Table 4.

The degree of overlap between the 5450 DMPs selected for WHSUS and the DMPs of other constitutional disorders from the previously published EpiSign v3 clinical classifier within EKD^13^ was visualized (Supplemental Figure 4). The highest overlap with WHSUS DMPs was observed in intellectual developmental disorder, autosomal dominant 23 (MRD23, OMIM 615761) and Kleefstra syndrome 1 (KLEFS1, OMIM 610253).

To explore the overall pattern of hyper- and hypomethylation among the WHSUS DMPs, the mean methylation difference between the case and control groups was calculated for each probe. The average of these values was computed and depicted in Supplemental Figure 5, where slight overall hypomethylation was observed for WHSUS. Similarly, the values for the DMPs identified in other disorders are also visualized in Supplemental Figure 5.

The degree of similarity in methylation levels between individuals with WHSUS and those with other syndromes was investigated using a tree-and-leaf diagram (Supplemental Figure 6). In this diagram, Phelan-McDermid syndrome (PHMDS, OMIM 606232) is positioned closest to the WHSUS cohort, indicating the highest degree of similarity in methylation patterns.

The DMPs and DMRs were annotated based on their positions relative to CpG islands and genes, with the results shown in Supplemental Figure 7. Around 50% of WHSUS DMPs were located within CpG islands or shores, compared with approximately 40% for the overall probe distribution. Additionally, close to 40% of WHSUS DMPs were found in gene promoters or promoter+ regions, whereas only about 25% of all probes fell within these regions.

### Gene set enrichment analysis

The GO enrichment analysis of the DMPs identified 3 significant terms using an adjusted *P* value threshold of 0.05. The details of these enriched terms and the significant genes associated with each are provided in Supplemental Table 5. These terms relate to various cellular processes and include numerous key genes from several families, such as solute carrier (SLC), collagen (COL), G protein-coupled receptor (GPR), cadherin (CDH), and ADAMTS (A Disintegrin and Metalloproteinase with Thrombospondin motifs).

## Discussion

Mendelian NDDs commonly present with complex and overlapping symptoms, which complicate clinical diagnosis. Individuals with various rare NDDs often exhibit unique disorder-specific DNA methylation patterns resulting from underlying gene defects. These DNA methylation profiles not only offer insights into the pathophysiology and molecular biology of these disorders but also serve as sensitive and specific diagnostic biomarkers.^31^ Episignatures detectable in peripheral blood provide easily accessible markers suitable for clinical settings. To establish episignatures as reliable diagnostic tools in clinical practice, it is essential to develop large reference databases and cohorts that have a shared genetic etiology and clinical phenotypes.

The identified episignature is sensitive and specific, capable of reclassifying VUS. Three of five VUS were reclassified as pathogenic based on the episignature results, despite conflicting *in silico* prediction scores (variants NC_000001.10:g.151378192A>C, NM_015100.4:c.3319T>G, p.(Phe1107Val); NC_000001.10:g.151380943G>T, NM_015100.4:c.2176C>A, p.(Pro726Thr); NC_000001.10:g.151377546G>A, NM_015100.4:c.3965C>T, p.(Ser1322Phe)). The 2 remaining VUS could not be reclassified. For P15, the variant was maternally inherited from an asymptomatic parent, and the patient's phenotype included features distinct from WHSUS, particularly within the marfanoid spectrum, in addition to NDD. For P16, the clinical presentation was significantly influenced by a history of extreme prematurity (25 weeks of gestation), with neuroimaging findings consistent with prematurity-related brain injury, providing a plausible alternative explanation for the observed NDD. These observations highlight the added value of episignature analysis in supporting the classification of VUS, particularly in cases where clinical presentations may be nonspecific. Discordance between *in silico* predictions and classification based on the episignature is expected, as *in silico* algorithms typically rely on factors such as the variant's presence in a conserved domain or amino acid properties, while methylation episignatures provide functional *in vivo* evidence of pathogenicity related to disrupted gene function. Overall, this approach enabled accurate diagnoses for 5 individuals.

Episignatures have been reported to correlate with clinical severity, with milder phenotypes sometimes showing milder methylation differences, although additional genetic and environmental factors likely contribute to this variability. A genotype–phenotype study of 117 individuals with *POGZ* variants, including 12 newly reported cases, found that missense variants were more often linked to milder presentations, whereas truncating variants predicted to escape NMD were associated with more severe outcomes.^2^ Variants involved in the proline-rich region showed the strongest association with severe phenotypes, suggesting potential gain-of-function or dominant-negative effects through NMD escape.^2^ In our methylation analysis, we used both supervised (SVM-MVP scoring) and unsupervised (hierarchical clustering and MDS) approaches to characterize sample relationships. Supervised scores indicate how confidently each sample aligns with the reference episignature, while unsupervised methods illustrate broader similarity patterns among individuals. These patterns can be influenced by factors such as sample size, the magnitude of methylation differences, and interindividual variability. Within this analytical framework, 5 individuals—P4, P5, P6, P8, and P13—exhibit comparatively higher MVP scores (Figure 2) and cluster together in the unsupervised analyses (Supplemental Figure 1). All 5 carry variants located within the NMD-escaping region proximal to the proline-rich domain (amino acids 851-1014; Nagy et al^2^) and preceding the CENP domain. Their subclustering therefore appears to reflect methylation differences associated with NMD-escaping variants occurring in this specific region of the gene rather than a pattern shared uniformly across all NMD-escaping variants.

Studies in *Pogz* knock-in mice have demonstrated that deficits in reciprocal social interaction were rescued with minimal doses of perampanel, a noncompetitive AMPA receptor antagonist. This rescue occurred by inhibiting the elevated neuronal excitability induced by the heterozygous *Pogz* pathogenic variant.^7^ The study evaluated both loss-of-function (*Pogz* knockdown) and overexpression of ASD-related *POGZ* mutants, demonstrating that these *de novo* variants impair neuronal migration through loss-of-function and dominant-negative effects. Perampanel has been established as well tolerated and effective in treating patients with drug-resistant epilepsy of unknown etiology. Furthermore, improvements in behavioral disturbances have been noted in patients treated with perampanel for refractory epilepsy, even when seizure control benefits were not observed.^32^, ^33^, ^34^, ^35^, ^36^ As a result, clinical trials for WHSUS are currently being discussed in Europe. This project presents an opportunity to explore potential changes in the WHSUS episignature as a therapeutic biomarker.

DMR analysis identified several regions overlapping genes with crucial roles, particularly in embryonic development. Notably, a predominantly hypomethylated region on chromosome 5, with a mean methylation difference >0.2, overlapped PR domain-containing protein 9 (*PRDM9*, OMIM 609760). *PRDM9* is a transcription factor with catalytic activity for the trimethylation of H3K4, which regulates the initiation and progression of meiosis.^37^ Another intriguing region is located on chromosome 4 and includes the gamma-aminobutyric acid receptor, gamma-1 (*GABRG1*, OMIM 137166), which belongs to the gamma-aminobutyric acid (GABA)-A receptor gene family. GABA receptors are essential proteins in GABAergic neurotransmission in the mammalian central nervous system.^38^ Among the regions of differential methylation, one overlaps with calcyphosine 2 (*CAPS2*, OMIM 607724), a gene encoding a secretory granule-associated protein that has previously been associated with cerebellar development and function in mouse models.^39^ While this observation does not suggest a direct causal relationship between CAPS2 methylation in blood and WHSUS neurological phenotypes, it may indicate systemic epigenetic dysregulation affecting developmental pathways. Such associations are hypothesis-generating and highlight candidate loci for future functional studies in relevant tissues.^40^ Importantly, we note that blood-derived episignatures may reflect systemic developmental disturbances rather than tissue-specific gene regulation, and gene-level observations should be interpreted cautiously and not as direct evidence of altered expression in affected tissues. Future studies integrating publicly available chromatin-binding datasets, such as *CHAMP1* and H3K9me3 ChIP-seq data from ENCODE, could help determine whether WHSUS-associated DMPs preferentially localize to *POGZ/CHAMP1/HP1α* heterochromatin sites. Such analyses could clarify the mechanistic link between *POGZ* haploinsufficiency, heterochromatin dysregulation, and the observed DNA methylation changes and potentially identify therapeutic molecular targets. Lastly, RBPJ-like protein (RBPJL, OMIM 616104), a paralog of RBPJ, is a transcription factor that plays a key role in the Notch signaling pathway. This pathway is crucial for making developmental decisions during embryonic and postnatal stages. It regulates differentiation across various organ systems, stem cell maintenance, and apoptosis.^41^ The GO enrichment analysis revealed more than 700 enriched genes related to numerous cellular processes essential for normal development. Among these, 40 genes belong to the solute carrier (SLC) family. These membrane transport proteins are crucial for transporting a diverse range of solutes, including sugars, amino acids, nucleotides, inorganic ions, and drugs.^42^ Additionally, 13 genes from the collagen (COL) superfamily were identified. These genes primarily provide structural support and strength to cells, maintaining biomechanical integrity across various tissues.^43^ Nine genes from the G protein-coupled receptor family were also found; these genes are involved in converting extracellular signals into significant physiological responses.^44^ Another 9 genes from the cadherin (CDH) family were detected, which encode transmembrane proteins important for cell-cell adhesion, tissue morphogenesis, and embryonic development.^45^ Lastly, 9 genes from the ADAMTS (A Disintegrin and Metalloproteinase with Thrombospondin motifs) family were recognized. These enzymes play diverse roles in tissue morphogenesis, extracellular matrix remodeling, and pathophysiological remodeling.^46^

In the functional correlation analysis, the heatmap provides a descriptive overview of the relative overlap of DMPs across different NDD episignatures (Supplemental Figure 4); however, the significance of these similarities has not been tested, and they should not be interpreted as evidence of shared molecular mechanisms. Any apparent overlap may simply reflect features commonly observed across NDDs rather than disorder-specific biological relationships. The tree-and-leaf diagram (Supplemental Figure 6) offers a more informative representation of the relative relationships among episignatures, illustrating general patterns without implying mechanistic similarity. While our DMR and GO analyses provide initial functional context, further functional analyses with larger sample sizes and pathway-specific functional studies will be required to clarify the biological significance of these overlaps and their potential mechanistic relationship to the clinical phenotype.

In conclusion, we have identified a novel DNA methylation episignature for WHSUS. This episignature demonstrates high sensitivity and specificity for *POGZ* haploinsufficiency, making it suitable for clinical diagnostics to identify and diagnose individuals with WHSUS, as well as to reclassify genetic VUS. Further research is needed to validate the diagnostic utility of this episignature in assessing *POGZ* missense variants, clinical severity, and monitoring therapeutics in clinical trials.

## Conflict of Interest

Brittany N. Simpson is a shareholder in EpiSign Inc, a biotech company involved in commercialization of EpiSign™ technology. The other authors declare no conflicts of interest.
